# Supplementary material for: Complexome profiling of the Chlamydomonas psb28 mutant reveals TEF5 as an early PSII assembly factor
Source: Plant Cell. 2025 Mar 17;37(6):koaf055. doi: 10.1093/plcell/koaf055 (PMC12164590; doi:10.1093/plcell/koaf055)
Supplement: koaf055_Supplementary_Data [file koaf055_supplementary_data.zip › psb28_interactive.zip!/plots/intensity/qualityPlots/peptides/OHP2_Cre06.g251150.t1.2.html]

### Cre06.g251150.t1.2

Trivial name: OHP2  
SeqCoverage [median]: 36.10  
Mass: 16.11 kDa  
  
 

| Sequence | MissedCleavage | Mass [Da] | wtIntensity | muIntensity | Score |
| --- | --- | --- | --- | --- | --- |
| LFGDASDASSPASTGAVTVEYQR | 0 | 2328.09 | 0.00 | 13306.15 | 134.29 |
| NEISNGR | 0 | 788.38 | 0.00 | 589.31 | 79.12 |
| QYFIDLR | 0 | 953.50 | 3805.83 | 5589.67 | 108.92 |
| QYFIDLRTEELR | 1 | 1581.82 | 650.09 | 0.00 | 40.59 |
| RLFGDASDASSPASTGAVTVEYQR | 1 | 2484.19 | 629.89 | 5641.40 | 108.64 |
| TQAFGWTPK | 0 | 1034.52 | 2327.29 | 0.00 | 117.01 |
|  |  |  | 7413.1 | 25126.5 |  |
